# The dynamic behaviors of complementary correlations under decoherence channels

**DOI:** 10.1038/srep40934

**Published:** 2017-01-30

**Authors:** Ming-Ming Du, Dong Wang, Liu Ye

**Affiliations:** 1 School of Physics & Material Science, Anhui University, Hefei, 230601, China

## Abstract

Complementary correlations can reveal the genuine quantum correlations present in a composite quantum system. Here, we explore an effective method to identify the entangled Bell diagonal states by means of Pearson correlation, one of the complementary correlations. Then, we extend this method to expose the dynamic behavior of complementary correlations under various kinds of decoherence channels. The sudden death and revival of entanglement can be explained by the idea of Pearson correlation. The threshold that is used to identify entanglement is proposed. Furthermore, we put forward a new method to expound the underlying physical mechanisms for which classical and quantum correlations suffer a sudden change in the decoherence process.

As is well known, quantum entanglement^1^ is a vital resource in quantum information processing (QIP) tasks, such as quantum teleportation^2^, quantum dense coding^3^, quantum remote preparation^4^,^5^,^6^, quantum cryptography^7^ and so on. Therefore, it has attracted a lot of attention. As a matter of fact, typical discussions of entanglement have been built on nonlocality, Bell inequality violations, monotones over local operations and classical communication, etc. For example, entanglement was discussed in many previous literatures focused on time reversal, local uncertainty relations^8^, entanglement witnesses^9^,^10^, entropic uncertainty relations^11^,^12^, concurrence^13^ and the covariance matrix criterion^14^. However, it is important to note that some states with zero entanglement can perform some tasks which are not possible in a classical regime. The conventional entangled-separate state framework seems to be inappropriate in the sense of characterizing and quantifying quantum correlations. Quantum correlation is more general and more fundamental than entanglement, which offers a more useful non-classical resource. Actually, the system is generally open and avoidably interacts with its surrounding environment^15^,^16^,^17^,^18^,^19^, resulting in a loss of quantum coherence, which in turn destroys the quantum correlations. Recently, the dynamics of quantum and classical correlations under both Markovian and non-Markovian decoherence have extensively observed^20^,^21^,^22^,^23^. Interestingly, contrary to the case of entanglement dynamics where sudden death might occur^24^,^25^,^26^, quantum correlation does not exhibit such a behavior.

Quantum physics differs significantly from classical physics in many aspects. A complete classical description of an object contains information concerning only compatible properties, while a complete quantum description of an object contains complementary information concerning incompatible properties. This difference also appears in correlations. Recently, Lorenzo Maccone *et al*.^27^ defined the complementary correlations, and entanglement detection based on which is capable of detection of a variety of entangled states that entanglement witnesses miss. And Wu *et al*.^28^ revealed that for a bipartite quantum state, the classical correlation is the maximal correlation based on a certain optimal basis, while the quantum correlation is characterized as a series of residual correlations in the mutually unbiased bases. Furthermore, Prasenjit Deb *et al*.^29^ optimized the previous outcomes. They explained what the underlying physical mechanisms are for classical and quantum correlations (measured in terms of discord) suffering sudden change in the decoherence process. The above paper, however, has used two complementary observables. It is well known that all systems have at least three complementary observables and there are *d* + 1 for d-dimensional systems if d is a power of a prime^30^. Motivated by this, our work is readily to extend to a general case of three complementary observables.

To begin with, we review different measures of complementary correlations, and then state our results. The detail proofs of the results are rendered in the part of Methods.

## Results

### Complementary correlations

Complementary correlaitions, introduced by Lorenzo Maccone *et al*.^27^, can reveal the genuine quantum correlations present in a composite quantum system. If the classical correlation is the maximal correlation based on a certain optimal basis, the quantum correlation is characterized as a series of residual correlations on the mutually unbiased bases. In other words, we can not only obtain classical correlation, but also can obtain the quantum correlation by residual correlations on the mutually unbiased bases.

**Figure 1:**
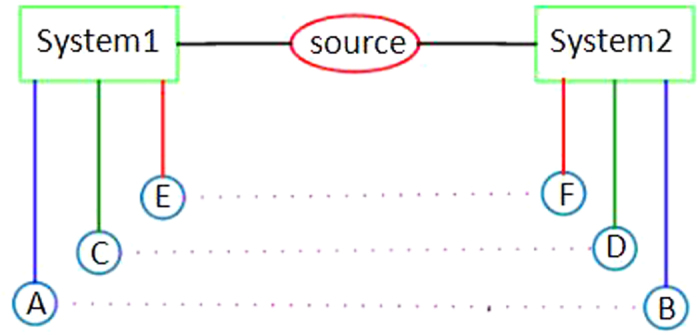
Complementary correlation measurements. System 1 and System 2 are the subsystems of the composite system. *A, C* and *E* are the complementary observables for system1 and *B, D* and *F* are the complementary observables for system 2.

Considering a quantum system with finite dimension *d*, let *M* and *N* be two observables acting on the system, with |*m*_*i*_〉 and |*n*_*i*_〉 denoting the non-degenerate eigenstates. If we have 
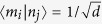
 for all *i, j*, where *d* is the dimension of Hilbert space, *M* and *N* are two complementary observables, respectively. According to the Supplement Material of ref. 27, for a bipartite system in a Hilbert space *H*_1_ ⊗ *H*_2_, there are three observable *A* ⊗ *B, C* ⊗ *D* and *E* ⊗ *F* (see Fig. 1), where *A, C* and *E* are complementary observables in the first system, *B, D* and *F* in the second. Then the quantity |*χ*_*AB*_| + |*χ*_*CD*_| + |*χ*_*EF*_| denotes the value of the complementary correlations, where |*χ*_*XY*_| denotes the absolute values of the correlations for complementary observables and can be quantified by any one of the three possibilities: Pearson correlation coefficient, mutual information and sum of conditional probabilities. In this paper, we special focus on Pearson correlation coefficient and mutual information.

### Pearson correlation

The Pearson correlation coefficient *C*_*XY*_ is defined as


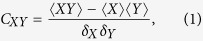


where *X* and *Y* denote observables relative to the two systems, 〈M〉 = *Tr*[*Mρ*] is the expectation value for the quantum state *ρ* and 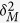 is the variance of the observable *M*. It is worth noting that the eigenstates of *X* or *Y* cannot apply to above quantity. The complementary correlation reads as *C*_*AB*_ + *C*_*CD*_ + *C*_*EF*_. For the correlation, one can obtain the following results^27^:

- *C*_*XY*_ = 0 for uncorrelated (product) states.
- If a state is maximally entangled if and only if there exist three complementary bases of linear observables such that *C*_*AB*_ + *C*_*CD*_ + *C*_*EF*_ = 3.
- If *C*_*AB*_ + *C*_*CD*_ + *C*_*EF*_ > 1, the two systems are entangled. It gives a sufficient condition for entanglement that can be used for entanglement detection.
- The separable states fulfill the condition *C*_*AB*_ + *C*_*CD*_ + *C*_*EF*_ ≤ 1.

### Mutual information

The mutual information *I* is defined by


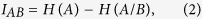


where *H (A*) = Σ_*a*_*p(a*) log_2_(*p(a*)) is the Shannon entropy of the measurement *A* performed on the first system, where *p(a*) is the probability of outcome *a*, and *H (A/B*) is the conditional entropy conditioning on the second system, which can be written as *H (A/B*) = Σ_*a*_*p(b)H (A/B* = *b*), where *H (A/B* = *b*) = −Σ_*a*_*p(a/b*) log_2_ *p(a/b*) is the entropy of the probability distribution *p(a/b*) for fixed outcome *b*. Therefore, in terms of mutual information, the complementary correlation reads as *I*_*AB*_ + *I*_*CD*_ + *I*_*EF*_. It can be easily shown that:

- The state of a bipartite quantum system is maximally entangled, if and only if there exist three complementary measurement bases, where *I*_*AB*_ + *I*_*CD*_ + *I*_*EF*_ = 3log *d*.
- If *I*_*AB*_ + *I*_*CD*_ + *I*_*EF*_ > log *d*, the state of the bipartite system is entangled.
- The separable states fulfill the condition *I*_*AB*_ + *I*_*CD*_ + *I*_*EF*_ ≤ log *d*.

### Identification of entangled Bell diagonal states

The density operator of two-qubit Bell diagonal states has the form


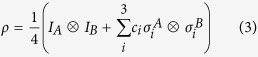


where *I*^*A(B*)^ is an identity matrix, *σ*_*i*_ denote Pauli matrices and *c*_*i*_ are spin-spin correlation functions. In matrix notation, we explicitly have


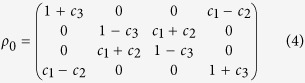


In this paper, we choose three suitable complementary observables, *σ*_1_, *σ*_2_, *σ*_3_ forming a set of complementary observables, which are used for a qubit and *A* = *B* = *σ*_1_, *C* = *D* = *σ*_2_, *E* = *F* = *σ*_3_. According to Eqs (1, 2 and 4), the classical correlations for complementary properties can be obtain as


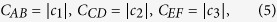


and


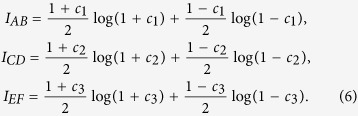


**Figure 2:**
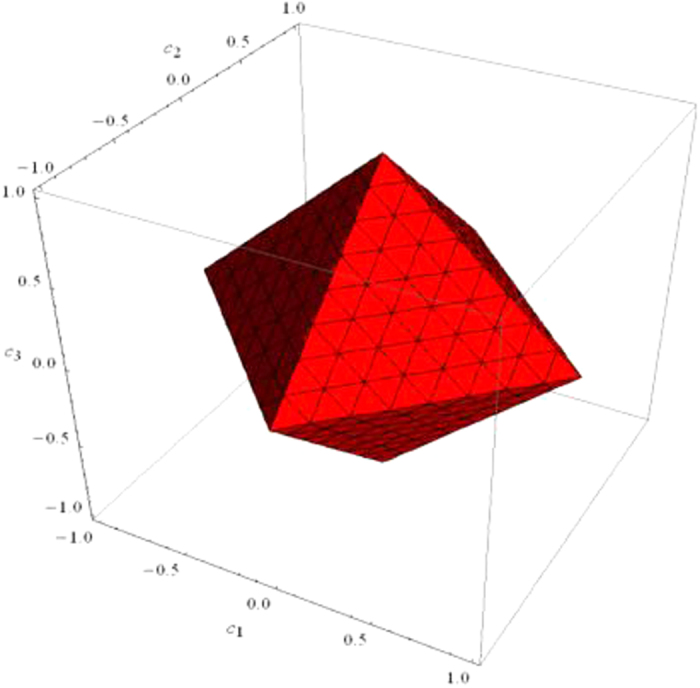
Geometrical representation of Bell-diagonal states. All Bell diagonal states lie in the tetrahedron *I*. Separable states are inside of octahedron (red region).

With respect to the four Bell states (|*c*_1_| = |*c*_2_| = |*c*_3_| = 1), it satisfies the conditions *C*_*AB*_ + *C*_*CD*_ + *C*_*EF*_ = 3. As to Werner states 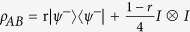, (|*c*_1_| = |*c*_2_| = |*c*_3_| = *r*), the state parameter *r* ∈ [0, 1], according to Eq. (4) and criterion (c1), it can be obtained that when 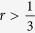, the system is entangled. Besides, we plot the Bell diagonal states as a function of *c*_1_, *c*_2_, and *c*_3_ in Fig. 2. From the figure, all Bell diagonal states lie in the tetrahedron *I*, with four vertices situate on the points of (1, 1, −1), (1, −1, 1), (−1, 1, 1), and (−1, −1, −1) being four Bell states (*C*_*AB*_ + *C*_*CD*_ + *C*_*EF*_ = 3). When *C*_*AB*_ + *C*_*CD*_ + *C*_*EF*_ > 1, there are four entangled regions that can be used as entanglement identification outside of red region. If the state is separable, it will surely satisfy *C*_*AB*_ + *C*_*CD*_ + *C*_*EF*_ ≤ 1, it is inside of red region. The red plane *C*_*AB*_ + *C*_*CD*_ + *C*_*EF*_ = 1 can be used as the entanglement threshold of Bell diagonal states. Compared with Concurrence, which is a measure of entanglement, Pearson correlation can be used to identify all two-qubit entangled Bell diagonal states. In the part of Methods, we present the strict numerical proof.

### The dynamic behavior of complementary correlation under the decoherence channels

Now, we will explore the influences of decoherence channels on the Bell diagonal states, when the states are coupled with local decoherence channels. In this context, we consider the system-environment interaction through the operator-sum representation formalism. Following the Kraus operators approach, the time-evolved state under local noisy environment can be described by the trace-preserving quantum operation, which is


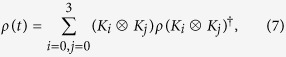


where *K*_*i,j*_ is Kraus operator corresponding decoherence channel, satisfying the trace-preserving condition 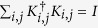. For the sake of simplicity, we provide a list of Kraus operators for a variety of channels considered in Table 1, where *p* is the time dependent parameter. As a matter of convenience, the dynamics evolutions of correlations with the time for the two-qubit states can be substituted by those with the parameter *p*. Decoherence processes (bit flip, phase flip and bit phase flip) preserve the Bell-diagonal form of the density operator *ρ*. In this situation, we can write the quantum operation *ε(ρ*) as


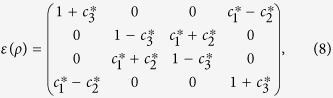


where 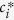 is shown in Table 2. Similarly, we can also give the entanglement critical condition that can be used for entanglement identification under decoherence channels by the threshold 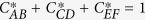. When *p* < p*, the entanglement is non-zero, vice versa, where p* is the entanglement critical value in Table 3.

Here we take two-qubit Bell diagonal states under the bit flip as an example to illustrate why entanglement suffers a sudden death.

The bit flip channel signifies that it flips both the bit and the phase of a qubit. The evolution of a density matrix *ρ* for it is given by


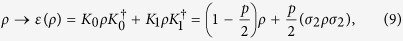


If one considers a single-qubit pure state *ψ*, then the evolution of the state for the bit-phase flip channel can be given by the mapping |*ψ*〉 → *σ*_2_|*ψ*〉.

According to Eqs (1) and (8), the Pearson correlation coefficients can be obtain as 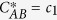, 
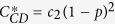
 and 
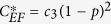
. From the numeral calculation, we can see that 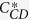 and 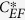 are degenerated, because of the effect of noisy channel. When 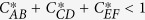, the entanglement undergoes a sudden death. In other words, entanglement degeneration is shown in the degeneration of 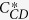 and 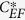. It can give us a new idea to protect entanglement by attempting protection of 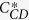 and 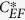.

### Sudden Transition between Classical and Quantum Correlation in the decoherence process

Sudden transition between classical and quantum correlation has been studied within several literatures^19^,^21^,^22^. In this paper, we will give a new interpretation based on complementary correlations.

We proceed by investigating the sudden transition of correlations using the Bell diagonal states. Furthermore, we consider the initial states as *c*_1_ = 1 and 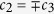, with *c*_3_ < 1. From the definition of Pearson correlation coefficient and mutual information, we have


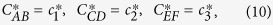


and


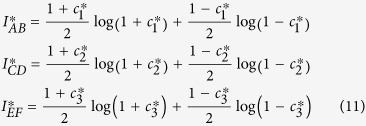


and the total complementary correlations are


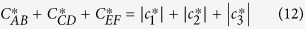


and


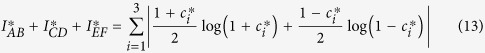


According to ref. 31, the classical correlation and quantum correlation, measured by geometric quantifiers, for Bell-diagonal states, are given by 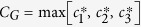 and 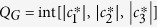, respectively, where 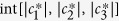 describes the intermediate result among the absolute values of the correlation functions 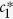, 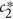, and 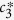. The classical correlation (J) and quantum correlation (QD)^21^, measured based on entropy, can be obtained


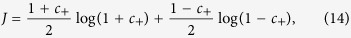


and


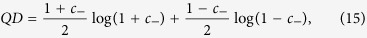


where 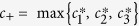 and 
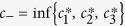
. With Eqs (10) and (11), we find that


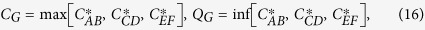


and


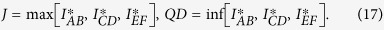


From above, they show that complementary correlation not only reveals the behavior of classical correlation, but also show the behavior of quantum correlation.

**Figure 3:**
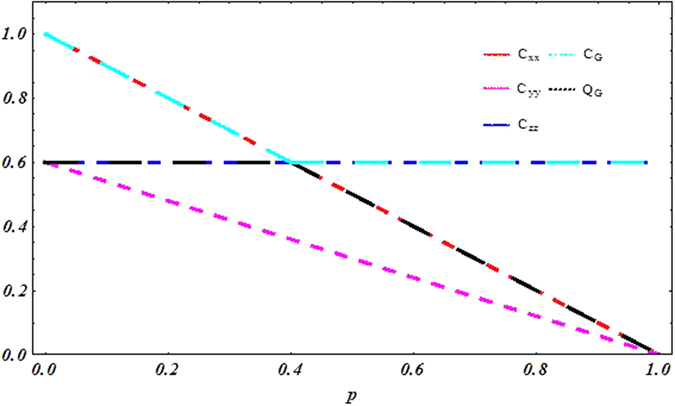
Sudden change behavior for the state given by *c*_1_ = 1 and *c*_2_ = −*c*_3_ = −0.6 under the phase flip channel.

**Figure 4:**
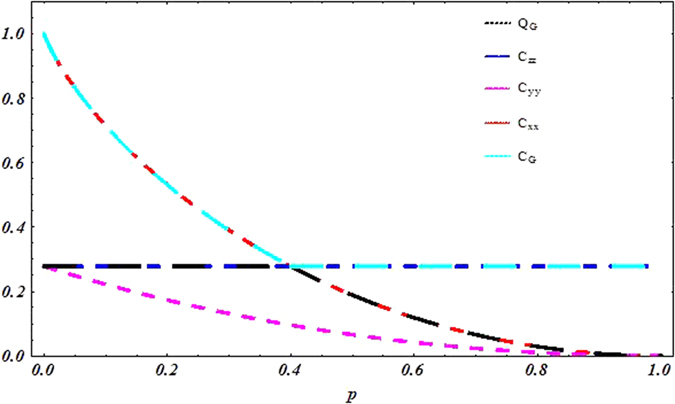
Sudden change behavior for the state given by *c*_1_ = 1 and *c*_2_ = −*c*_3_ = −0.6 under the phase flip channel.

To address the question about sudden transition between classical and quantum correlation under local decoherence channels, we focus on exploring dynamical behavior of quantum correlations under phase flip channel. Conveniently, we depict the behavior of Pearson correlation coefficient, geometric classical correlation, geometric quantum discord with the parameter *p* for *c*_1_ = 1 and *c*_2_ = −*c*_3_ = −0.6 in Fig. 3 and the behavior of mutual information, classical correlation and quantum discord in Fig. 4. These plots clearly display the sharp transition from classical to quantum correlation occurring at *p* = *p*^sc^ in decoherence regime. When 0 < *p* < p^*sc*^, the classical correlations decay and the quantum correlations remain constant. In other words, we can say that the correlations 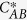 and 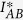 decay, the correlations 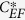 and 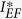 remain constant. When *p* > *p*^*sc*^, the classical correlation is constant, whereas the quantum correlation starts to decay. Analyzing physical origin of the sudden transition, we consider that when 0 < *p* < *p*^*sc*^, the projective measurement on the eigenstates of observables *σ*_1_ will yield the classical correlations, the quantum correlation is characterized in the eigenstates of observables *σ*_3_. Corresponding, when *p* > *p*^*sc*^ the projective measurement on the eigenstates of observables *σ*_3_ will yield the classical correlations, the quantum correlation is characterized in the eigenstates of observables *σ*_1_. Meanwhile, we notice that correlation is constant under this processing, its physical source is that the phase flip channel induces a decay in the quantum coherence of the state *ε(ρ*), which results in a decay in the expectation of *σ*_1_ ⊗ *σ*_1_, whereas the expectation value of *σ*_3_ ⊗ *σ*_3_ remains constant. If we consider, instead of bit flip noise, the bit-phase flip channel, then the coefficients 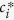 change as Table 2. It is very easy to exhibit the same sudden transition in their evolution. Moreover, such sudden transitions can also be explained through complementary correlations.

## Conclusions

To summarize, we explore an effective method to identify the entangled Bell diagonal states by means of Pearson correlation, one of the complementary correlations. Furthermore, the dynamic behavior of complementary correlations under various kinds of decoherence channels are exposed. A new interpretation of sudden death and revival of entanglement in terms of Pearson correlation is proposed. The entanglement threshold p* being used to identify entanglement is revealed. Meanwhile, we find that the dynamic behavior of classical correlations for complementary properties is a good interpretation in quantum correlation problem. If the classical correlation is the maximal correlation on a certain optimum basis, the quantum correlation will be characterized as a series of residual correlations on the mutual unbiased bases. In other words, the sudden transition behaviors reveal not only classical but also quantum correlations in terms of complementary correlations. Therefore, we believe our investigation might bring some new attempts to study protection of entanglement and provide a nice insight into interpreting sudden transition behavior of classical and quantum correlations in the decoherence process.

## Methods

### Entangled Bell diagonal states

The entanglement of Bell-diagonal states coupled with the noisy environments can be quantified conveniently by concurrence^25^, *E* = max{0, 2 max *λ*_*i*_ − 1}, where *λ*_*i*_ is the eigenvalue of Bell-diagonal states. We can obtain that the state is entangled when 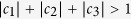 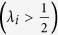.

### Geometric correlations

Geometric quantifiers of quantum (namely, GQD-1) and classical correlations between A and B can be defined through the trace distances^31^, *Q*_*G*_ = inf tr|*ρ* − *χ*| = tr|*ρ* − *χ*_*ρ*_| and *C*_*G*_(*ρ*) = tr|*χ*_*ρ*_ − *π*_*ρ*_|, where *χ*_*ρ*_ is a classical-quantum state and *π*_*ρ*_ represents the product of the local marginals of *ρ*.

### Classical correlation and quantum discord

The classical correlations of a composite quantum state can be quantified via the measure proposed by Henderson and Vedral^32^ which is given by 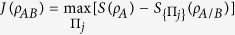, where the maximum is taken over the set of projective measurements {∏_*j*_} on subsystem *B*. 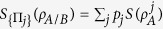 is the entropy of subsystem *A* conditioned on *B*, 
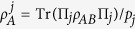
 is the density matrix of subsystem *A* depending on the measurement outcome for *B*, and 
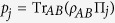
 is the probability of the *jth* outcome.

The quantum discord, which is a measure of quantum correlation, is defined as *QD(ρ*_*AB*_) = *I(ρ*_*AB*_) − *J(ρ*_*AB*_), where *I(ρ*_*AB*_) = *S(ρ*_*A*_) + *S(ρ*_*B*_) − *S(ρ*_*AB*_) is the quantum mutual information.

## Additional Information

**How to cite this article**: Du, M.-M. *et al*. The dynamical behaviors of complementary correlations under decoherence channels. *Sci. Rep.* **7**, 40934; doi: 10.1038/srep40934 (2017).

**Publisher's note:** Springer Nature remains neutral with regard to jurisdictional claims in published maps and institutional affiliations.

## Figures and Tables

**Table 1:** The Kraus operators for the quantum channels: bit flip, phase flip, and bit-phase flip channels.

Channel	Kraus operators	
Bit flip	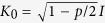	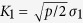
phase flip	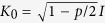	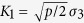
bit-phase flip	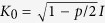	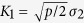

**Table 2:** Correlation functions for the quantum operations: bit flip, phase flip and bit-phase flip channels.

Channel	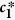	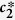	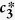
Bit flip	*c*_1_	*c*_2_(1 − *p*)^2^	*c*_3_(1 − *p*)^2^
phase flip	*c*_1_(1 − *p*)^2^	*c*_2_(1 − *p*)^2^	*c*_3_
bit-phase flip	*c*_1_(1 − *p*)^2^	*c*_2_	*c*_3_(1 − *p*)^2^

**Table 3:** The entanglement critical value under the decoherence channels.

Channel	Entanglement critical condition p*
Bit flip	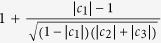
phase flip	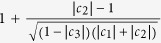
bit-phase flip	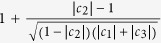

## References

[b1] Horodecki R., Horodecki P., Horodecki M. & Horodecki K. Quantum entanglement. Rev. Mod. Phys. 81, 865 (2009).

[b2] Manzoor I., Shi-Yao Z. & Suhail Zubairy M. Quantum teleportation of an entangled state. Phys. Rev. A 62, 022307 (2000).

[b3] Hyuk-jae L., Doyeol A. & Sung W. H. Dense coding in entangled states. Phys. Rev. A 66, 024304 (2002).

[b4] Bennett C. H., DiVincenzo D. P., Shor P. W., Smolin J. A., Terhal B. M. & Wootters W. K. Remote state preparation. Phys. Rev. Lett. 87, 077902 (2001). 11497918 10.1103/PhysRevLett.87.077902

[b5] Wang D. . Efficient and faithful remote preparation of arbitrary three- and four-particle W-class entangled states. Quantum Inf. Process. 14, 2135–2151 (2015).

[b6] Wang D. . Practical single-photon-assisted remote state preparation with non-maximally entanglement. Quantum Inf. Process. 15, 3367–3381 (2016).

[b7] Alexios B., Rosa B., Thierry G., André V., Jean-Philippe P. & Philippe G. Single Photon Quantum Cryptography. Phys. Rev. Lett. 89, 187901 (2002). 12398636 10.1103/PhysRevLett.89.187901

[b8] Gühne O., Mechler M., Tóth G. & Adam P. Entanglement criteria based on local uncertainty relations are strictly stronger than the computable cross norm criterion. Phys. Rev. A 74, 010301 (R) (2006).

[b9] Gühne O. & Tóth G. Entanglement detection. Phys. Rep. 474, 1 (2009).

[b10] Lewenstein M., Kraus B., Cirac J. I. & Horodecki P. Optimization of entanglement witnesses. Phys. Rev. A 62, 052310 (2000).

[b11] Giovannetti V. Separability conditions from entropic uncertainty relations. Phys. Rev. A 70, 012102 (2004).

[b12] Coles P. J. & Piani M. Improved entropic uncertainty relations and information exclusion relations. Phys. Rev. A 89, 022112 (2014).

[b13] Wootters W. K. Entanglement of Formation of an Arbitrary State of Two Qubits. Phys. Rev. Lett. 80, 2245 (1998).

[b14] Gühne O., Hyllus P., Gittsovich O. & Eisert J. Covariance Matrices and the Separability Problem. Phys. Rev. Lett. 99, 130504 (2007). 17930568 10.1103/PhysRevLett.99.130504

[b15] Mirza Imran M. & John C. S. Multiqubit entanglement in bidirectional-chiral-waveguide QED. Phys. Rev. A 94, 012302 (2016).

[b16] Mirza Imran M. & John C. S. Two-photon entanglement in multiqubit bidirectionalwaveguide QED. Phys. Rev. A 94, 012309 (2016).

[b17] Mirza Imran M., van Enk S. J. & Kimble H. J. Single-photon time dependent spectra in coupled cavity arrays. J. Opt. Soc. Am. B, 10, 2640–2649 (2013).

[b18] Mirza Imran M. & van Enk S. J. How Nonlinear Optical Effects Degrade Hong-Ou-Mandel Like Interference. Opt. Comm. 343, 172–177 (2015).

[b19] Maziero J., Céleri L. C., Serra R. M. & Vedral V. Classical and quantum correlations under decoherence. Phys. Rev. A 80, 044102 (2009).

[b20] Maziero J., Werlang T., Fanchini F. F., Céleri L. C. & Serra R. M. System-reservoir dynamics of quantum and classical correlations. Phys. Rev. A 81, 022116 (2010).

[b21] Montealegre J. D., Paula F. M., Saguia A. & Sarandy M. S. One-norm geometric quantum discord under decoherence. Phys. Rev. A 87, 042115 (2013).

[b22] Mazzola L., Piilo J. & Maniscalco S. Sudden transition between classical and quantum decoherence Phys. Rev. Lett. 104 200401 (2010). 20867012 10.1103/PhysRevLett.104.200401

[b23] Haikka P., Johnson T. H. & Maniscalco S. Non-Markovianity of local dephasing channels and time-invariant discord. Phys. Rev. A 87, 010103 (2013).

[b24] Aolita L. . Scaling laws for the decay of multiqubit entanglement. Phys. Rev. Lett. 100, 080501 (2008). 18352609 10.1103/PhysRevLett.100.080501

[b25] Yu T. & Eberly J. H. Sudden death of entanglement. Science 323, 598 (2009). 19179521 10.1126/science.1167343

[b26] Almeida M. P. . Environment-induced sudden death of entanglement. Science. Science 316, 579 (2007). 17463284 10.1126/science.1139892

[b27] Maccone L., Bruß D. & Macchiavello C. Complementary and correlations. Phys. Rev. Lett. 114, 130401 (2015). 25884117 10.1103/PhysRevLett.114.130401

[b28] Wu S., Ma Z., Chen Z. & Yu S. Reveal quantum correlation in complementary bases. Sci. Rep. 4, 4036 (2014). 24503595 10.1038/srep04036 PMC3916891

[b29] Dep P. & Banik M. Role of complementary correlations in the evolution of classical and quantum correlations under Markovian decoherence. J. Phys. A: Math. Theor. 48, 185303 (2015).

[b30] Durt T., Englert B. G., Bengtsson I. & Życzkowski K. On mutually unbiased bases. Int. J. Quantum. Inform. 08, 535 (2010).

[b31] Paula F. M., Silva I. A., Montealegre J. D., Souza A. M., de Azevedo E. R., Sarthour R. S., Saguia A., Oliveira I. S., Soares-Pinto D. O., Adesso G. & Sarandy M. S. Observation of Environment-Induced Double Sudden Transitions in Geometric Quantum Correlations. Phys Rev Lett. 111, 250401 (2003). 10.1103/PhysRevLett.111.250401 24483731

[b32] Henderson L. & Vedral V. Classical, quantum and total correlations. J. Phys. A: Math. Gen 34, 06899 (2001).

